# Regulation and Role of αE Integrin and Gut Homing Integrins in Migration and Retention of Intestinal Lymphocytes during Inflammatory Bowel Disease

**DOI:** 10.4049/jimmunol.2100220

**Published:** 2021-11-01

**Authors:** Mary E. Keir, Franklin Fuh, Ryan Ichikawa, Meghan Acres, Jason A. Hackney, Gillian Hulme, Christopher D. Carey, Jeremy Palmer, Claire J. Jones, Anna K. Long, Jenny Jiang, Sha Klabunde, John C. Mansfield, Cary M. Looney, William A. Faubion, Andrew Filby, John A. Kirby, Jacqueline McBride, Christopher A. Lamb

**Affiliations:** * Genentech Inc., South San Francisco, CA;; † Translational and Clinical Research Institute, Biosciences Institute, Faculty of Medical Sciences, Newcastle University, Newcastle upon Tyne, United Kingdom;; ‡ Department of Histopathology, Newcastle upon Tyne Hospitals NHS Foundation Trust, Newcastle upon Tyne, United Kingdom;; § Flow Cytometry Core Facility and Innovation, Methodology and Application Research Theme, Biosciences Institute, Faculty of Medical Sciences, Newcastle University, Newcastle upon Tyne, United Kingdom;; ¶ Department of Haematology, Newcastle upon Tyne Hospitals NHS Foundation Trust, Newcastle upon Tyne, United Kingdom;; ‖ Department of Gastroenterology, Newcastle upon Tyne Hospitals NHS Foundation Trust, Newcastle upon Tyne, United Kingdom; and; # Mayo Clinic, Rochester, MN

## Abstract

Targeting interactions between α4β7 integrin and endothelial adhesion molecule MAdCAM-1 to inhibit lymphocyte migration to the gastrointestinal tract is an effective therapy in inflammatory bowel disease (IBD). Following lymphocyte entry into the mucosa, a subset of these cells expresses αEβ7 integrin, which is expressed on proinflammatory lymphocytes, to increase cell retention. The factors governing lymphocyte migration into the intestinal mucosa and αE integrin expression in healthy subjects and IBD patients remain incompletely understood. We evaluated changes in factors involved in lymphocyte migration and differentiation within tissues. Both ileal and colonic tissue from active IBD patients showed upregulation of ICAM-1, VCAM-1, and MAdCAM-1 at the gene and protein levels compared with healthy subjects and/or inactive IBD patients. β1 and β7 integrin expression on circulating lymphocytes was similar across groups. TGF-β1 treatment induced expression of αE on both β7^+^ and β7^−^ T cells, suggesting that cells entering the mucosa independently of MAdCAM-1/α4β7 can become αEβ7^+^. ITGAE gene polymorphisms did not alter protein induction following TGF-β1 stimulation. Increased phospho-SMAD3, which is directly downstream of TGF-β, and increased TGF-β–responsive gene expression were observed in the colonic mucosa of IBD patients. Finally, in vitro stimulation experiments showed that baseline β7 expression had little effect on cytokine, chemokine, transcription factor, and effector molecule gene expression in αE^+^ and αE^−^ T cells. These findings suggest cell migration to the gut mucosa may be altered in IBD and α4β7^−^, and α4β7^+^ T cells may upregulate αEβ7 in response to TGF-β once within the gut mucosa.

## Key Points

- Adhesion molecules are upregulated in inflamed intestinal mucosa in IBD patients.
- Baseline β7 expression does not impact αE induction or gene expression in T cells.
- Phospho-SMAD3 is increased in inflamed mucosa in IBD.

## Introduction

The immune system relies on a complex network of mechanisms to enable immune surveillance of tissues. Migration of leukocytes into intestinal tissues involves chemokines, adhesion molecules, and integrins to mediate rolling along the endothelium, arrest following integrin activation, and binding to adhesion molecules and transmigration (1). Circulating T cells in the peripheral blood can enter the gut through an endothelial barrier in one of two ways: 1) naive T cells migrate to gut-associated lymphoid tissue or 2) memory T cells previously activated in gut-associated lymphoid tissue enter the lamina propria (LP) via systemic circulation (2). Under normal homeostatic conditions, migration into the gut occurs through interactions between α4β7 integrin expressed on both naive and memory T cells and MAdCAM-1 on the endothelium (2, 3). To a lesser extent, cells are thought to use α4β1 (very late Ag 4 [VLA-4]) interacting with vascular adhesion molecule-1 (VCAM-1) and fibronectin (4), or αLβ2 (lymphocyte function-associated Ag) interacting with ICAM-1 to migrate to the intestine (5, 6). Recent data suggest that α4β1 may be particularly important for migration to the small intestine in comparison with the colon (7). Once lymphocytes have entered the intestinal mucosa, a subset of T cells upregulates αEβ7 (CD103) (8). αEβ7 binds to E-cadherin and increases the retention of cells in the intestinal mucosa (9).

Aberrant innate and adaptive mucosal immune responses to microbes in the gut lumen characterize both Crohn’s disease (CD) and ulcerative colitis (UC), the two types of inflammatory bowel disease (IBD) (10). During inflammation, intestinal vascular endothelium can be activated by inflammatory cytokines, including TNF-α and IL-1β, and other stimuli, such as LPS, to upregulate expression of VCAM-1 and ICAM-1 (11), which have the potential to alter normal lymphocyte migration patterns into the intestine. Early studies in IBD showed that MAdCAM-1 is upregulated in IBD samples (12), whereas results for VCAM-1 and ICAM-1 are mixed (13, 14). Investigating inflammation-induced changes in lymphocyte migration is important for understanding current anti-integrin therapies for IBD that target lymphocyte migration to the intestine.

Differences between blockade of α4 integrin (natalizumab) (15), α4β7 integrin heterodimer (vedolizumab) (16, 17), and β7 integrin (etrolizumab) (18, 19), which blocks both α4β7 and αEβ7 heterodimers, may be key to understanding differences in therapeutic efficacy. Both α4β7 and α4β1 are expressed by a large proportion of circulating T cells (20). αEβ7, in contrast, is expressed by very few circulating T cells but is highly expressed by intestinal T cells both in the LP and intraepithelial space (21). TGF-β1 is a multifunctional cytokine present in the intestinal mucosa that can signal to T cells, is associated with commitment to the Th17 cell lineage, and also induces αE expression. Disease-associated risk alleles in *ITGAE*, the gene that encodes αE integrin, have been associated with differential levels of surface expression of αE following TGF-β1 stimulation in vitro (22). It is unclear if circulating T cells require pre-existing expression of α4β7 integrin to express αEβ7 integrin in the gut, perhaps by switching the pairing of the α-subunit on β7^+^ cells from α4 to αE through increased αE surface expression. This is of particular importance, as data from our group and others suggest that a subset of potentially pathogenic T cells expresses αEβ7 integrin in UC (21, 23, 24).

The present study aims clearly to define inflammation-induced changes in lymphocyte migration that are relevant to interpreting clinical data for therapies that inhibit integrin-mediated lymphocyte migration to the intestine. Blockade of α4β7/MAdCAM-1 interaction has been shown to reduce lymphocyte trafficking. As we show in this study, induction of αEβ7 integrin on α4β7^−^ cells is possible, suggesting that cells trafficking to the gut via inflammation-induced alternative mechanisms (e.g., via α4β1/VCAM-1 or αLβ2/ICAM-1) may become αEβ7^+^ effector T cells. Our findings support both altered adhesion molecule patterns in the intestine in active IBD as well as differentiation of αE^+^ T cells from cells that initially enter the intestinal mucosa by non-α4β7/MAdCAM-1 means.

## Materials and Methods

### Human subjects

Healthy volunteers and IBD patients were enrolled in separate studies at Newcastle University, and in a previously described Mayo Clinic (25) and multicenter observational IBD study (Emerging Biomarkers [EMBARK]) (26). Patient characteristics for all cohorts are shown in Supplemental Table I. In addition, peripheral blood and PBMCs were collected from healthy donors participating in the Genentech blood donor program with written, informed consent. Saliva was collected from Genentech donors and genotyped on the Global Screening Array (Illumina, San Diego, CA) and confirmed by targeted PCR genotyping.

For the Newcastle cohort, none of the IBD patients were taking any medications for their disease, including biologics, immunomodulators, steroids, or 5-ASA. Active disease in the ileum or distal colon was defined as endoscopically visible mucosal inflammation and active inflammation was confirmed via histological examination by a pathologist. Inactive disease in the ileum or distal colon was defined as absence of visible inflammation at endoscopy and absence of histological inflammation by pathologist examination.

### Ethics

Written informed consent was obtained in accordance with research and ethics committee (REC) (Newcastle and North Tyneside 1 REC 10/H0906/41 and Newcastle and North Tyneside 2 REC 22/02; Mayo Clinic Institutional Review Board 10-006628) approval. These studies were performed according to the principles of the Declaration of Helsinki. The Western Institutional Review Board provided research and ethics oversight for the Genentech blood donor program and the EMBARK cohort has been previously described (26).

### Microarray

RNA was isolated as previously described (23). Microarray data (Gene Expression Omnibus https://www.ncbi.nlm.nih.gov/geo; accession number GSE 179285) were preprocessed and normalized using packages from the Bioconductor project (http://bioconductor.org), supplemented with scripts written in the R programming language (http://r-project.org). Feature-extracted data were background subtracted using the normal + exponential method from the limma Bioconductor package (27). Log-ratios for the test and reference channel were normalized using loess normalization (28). Datasets were independently normalized across samples using quantiles normalization (29). We filtered out probes from the microarrays that did not map to known Entrez Genes, and further reduced the data by selecting a single probe per gene, selecting the gene with the highest interquartile range as the exemplar for a given gene (30). Individual genes were plotted for each patient sample in the EMBARK cohort (26).

### Immunohistochemistry

Staining was performed using a Discovery Ultra Autostainer (Ventana). For each immunohistochemistry (IHC) Ab, positive control tissue was initially used for optimization and identification of staining morphology, typically with archival paraffin-embedded tonsil, thymus, bone marrow, or spleen. Optimal Ag retrieval, primary Ab dilution, and detection kits were then determined by staining 4-µm sections of intestine. All Ag retrieval was optimal using a Standard Cell Conditioning 1 buffer (Ventana) retrieval protocol for 64 min at 100°C. The following anti-human Abs were optimized: anti-pSMAD3 (phosphor-S423 and S425, rabbit monoclonal clone EP823Y, dilution 1:100; Abcam), anti–MAdCAM-1 (mouse monoclonal, clone 355G8; Thermo Fisher Scientific, dilution 1:100), anti–ICAM-1 (rabbit monoclonal, clone EPR4776, dilution 1:400; Abcam), and anti–VCAM-1 (rabbit monoclonal, clone EPR5047, dilution 1:250; Abcam). The Ultraview DAB Detection Kit (Ventana) was used for all Abs. Digital images were acquired using a Vectra 3 Slide Scanner (PerkinElmer) for cell adhesion molecules and an BX43 microscope (Olympus) for pSMAD3 IHC. Unless otherwise noted, representative images were selected from 10 samples stained in each group.

### Flow cytometry

Expression of integrin β7, β1, and αE on CD3^+^ peripheral homing (CD45RA^–^β7 low), mucosal homing (CD45RA^–^β7 high), and naive (CD45RA^+^β7 intermediate) T cell subsets were assessed by flow cytometry. In brief, whole blood samples from Mayo Clinic UC, CD, and non-IBD patients were collected into sodium heparin vacutainer tubes and shipped overnight at ambient temperature to Genentech. To lyse the RBCs, 1× BD Pharm Lyse buffer (BD Biosciences, San Jose, CA) was added to the whole blood sample and allowed to incubate at room temperature for 20 min. Samples were washed three times with ice cold staining buffer (PBS + 2% FBS) followed by addition of normal mouse serum to block potential nonspecific staining. Samples were incubated on ice for 20 min prior to staining aliquots with Ab mixtures containing fluorescently conjugated anti-CD3 (SP34-2), CD45RA (HI100), β7 (FIB504), β1 (MAR4), αE (Ber-ACT8), and/or the respective isotype controls (BD Biosciences, San Jose, CA). Samples were incubated on ice for 30 min, washed twice with staining buffer, and resuspended in 1% paraformaldehyde prior to acquisition on a BD LSR (BD Biosciences, San Jose, CA). A total of 75,000–100,000 lymphocyte gated events were acquired per sample. Flow cytograms were generated to establish the fraction of cells positive for each surface marker. Geometric mean fluorescent intensity (MFI) and percentage of gated cells for each T cell subset were assessed and reported accordingly.

### In vitro cell culture experiments

T cells were purified from PBMCs by immunomagnetic negative selection using the EasySep Human T Cell Enrichment Kit (STEMCELL Technologies) and cultured with CD3/CD28 Dynabeads (Invitrogen) in complete RPMI medium-1640 (RPMI) containing 10% FCS at a 1:1 cell/bead ratio with or without 10 ng/ml TGF-β1 (R&D Systems). In some experiments, an ALK5 inhibitor (SB-505124; Sigma-Aldrich) was included. For CFSE dilution experiments, purified T cells were labeled with 10 μM CFSE, stimulated at a 2:1 cell/bead ratio, and harvested at times indicated.

### In vitro stimulation of peripheral blood T cells

Frozen PBMCs were thawed with RPMI 1640 with 20% FCS, 50 IU/ml penicillin, 50 µg/ml streptomycin, 2 mM glutamax, and 25 U/ml benzonase (Sigma-Aldrich, St. Louis, MO) and rested overnight in 48-well culture plates in the incubator at 37°C. The next day, anti-CD3/CD28-coated Dynabeads (Thermo Fisher Scientific, Waltham, MA) were prepared per manufacturer instructions, resuspended in RPMI, and added to cells along with 5 ng/ml of recombinant TGF-β1 (Cell Signaling Technologies, Danvers, MA), 30 U/ml of recombinant IL-2 (PeproTech, Cranbury, NJ) and, where indicated, in the presence of 20 μg/ml etrolizumab (anti-β7), vedolizumab (anti-α4β7) or human IgG1 isotype control. After 6 d of stimulation, cells were harvested and stained. Viability was assessed using the LIVE/DEAD Fixable Near-IR Dead Cell Stain Kit (Thermo Fisher Scientific, Waltham, MA). Cells were blocked with human TruStain Fc block (BioLegend, San Diego, CA), incubated on ice for 10 min, stained with an Ab mixture of anti–CD8-V500 (SK1), anti–CD3-FITC (UCHT1), anti–CD103-PE (Ber-ACT8), anti–CD4-PerCP-Cy5.5 (SK3) (BD Bioscience, San Jose, CA), and were added for 20 min. In some experiments, β7 surface expression was assessed by staining with anti-β7 FIB504 allophycocyanin (BD Biosciences, San Jose, CA) or anti-β7 9D8 Alexa647 (Genentech, South San Francisco, CA). After washing, cells were resuspended in 1% paraformaldehyde (Electron Microscopy Sciences, Hatfield, PA) prior to analysis on a Canto II Flow Cytometer (BD Bioscience, San Jose, CA).

### Comparison of gene expression in T cells with or without baseline β7 expression

PBMCs from seven donors were phenotyped and sorted using a BD FACS Aria Fusion cell sorter (BD Biosciences) into CD3^+^β7^−^αE^−^ and CD3^+^β7^+^αE^−^ populations (Abs: anti-CD3 clone OKT3, anti-β7 clone Fib504, anti-αE clone Ber-ACT8, anti-αL clone HI111, anti-β1 clone TS2/16). A total of 5 × 10^5^ cells were cultured in 1 ml of complete RPMI (20% FCS) supplemented with 10 ng/ml TGF-β1 in a 1:1 ratio with anti-CD3/CD28 Dynabeads. Media was changed every 2–3 d with FCS reduced to 10% at the first media change. Following 10 d of culture, four cell populations, CD4^+^αE^+^, CD4^+^αE^−^, CD8^+^ αE^+^, and CD8^+^αE^−^ (Abs: anti-CD4 clone RPA-T4, anti-CD8 clone HIT8a), cultured from the β7^+^ and β7^−^ progenitor populations were sorted using a BD FACS Aria Fusion Cell Sorter into 1% 2-ME RLT RNeasy lysis buffer (Qiagen). RNA was extracted using RNeasy Kits (Qiagen). Fifty-six total tubes were sorted based on the original β7 surface expression in combination with CD4, CD8, and αE expression at the end of culture. Eight of these tubes had <1000 cells per group and two failed to yield enough RNA for analysis. First-strand cDNA was generated from 34 ng of total RNA using iScript (Bio-Rad Diagnostics, Hercules, CA). cDNA (2.125 ng) was preamplified using the TaqMan PreAmp Master Mix (Life Technologies, Carlsbad, CA) along with diluted TaqMan assays (Life Technologies, Carlsbad, CA), according to the manufacturer’s instructions. Quantitative PCR was performed using the Biomark HD system (Fluidigm 96.96 format).

### Comparison of selected genes in sorted in vitro differentiated cells

A linear model was used to evaluate the effect of baseline expression of β7 integrin on expression of selected genes following 10 d of culture with or without TGF-β1 stimulation as described. Both fold change and statistical significance was determined for all genes tested and a false discovery rate (FDR) was calculated using the method of Benjamini and Hochberg (31). The analysis was performed independently for CD4^+^ and CD8^+^ T cells, with day 10 αE integrin expression included as a covariate.

## Results

### Adhesion molecules ICAM-1, VCAM-1, and MAdCAM-1 are upregulated in IBD

**FIGURE 1.**
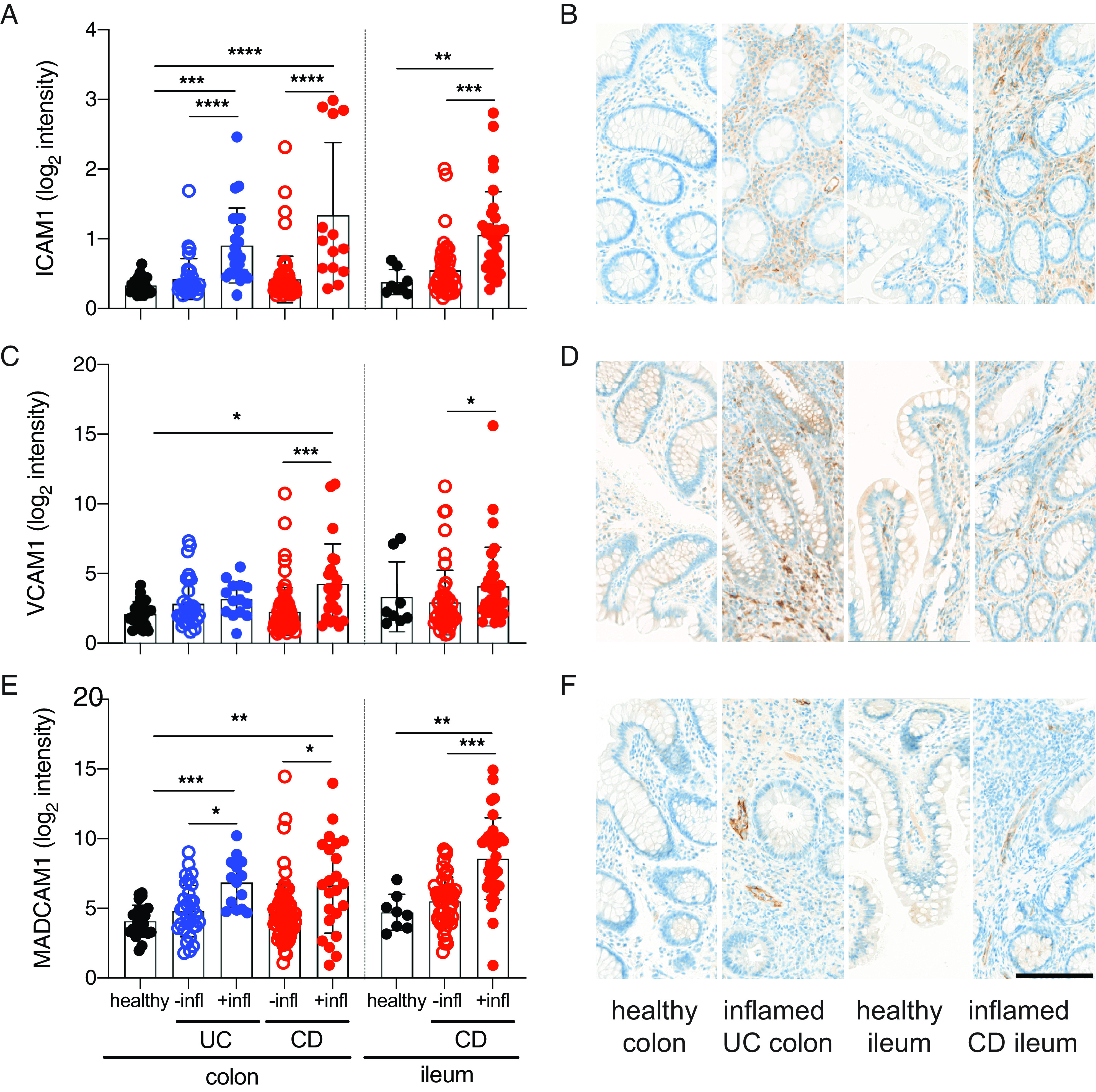
Upregulation of adhesion molecules ICAM-1, VCAM-1, and MAdCAM-1 in IBD. Gene expression and IHC of adhesion molecules in intestinal biopsy samples from healthy subjects and IBD patients from the EMBARK and Newcastle cohorts. (**A**) ICAM-1 gene expression levels in normal mucosal biopsies from healthy subjects (black), uninflamed (open circles), and inflamed (closed circles) biopsies from the colon and ileum. (**B**) Examples of ICAM-1 protein localization by IHC in (left to right) healthy colon, inflamed UC colon, healthy ileum, and inflamed CD ileum. (**C**) VCAM-1 gene expression and (**D**) protein localization by IHC in inflamed and uninflamed biopsies from the colon and ileum of healthy subjects and IBD patients. (**E**) MAdCAM-1 gene expression and (**F**) protein localization by IHC in inflamed and uninflamed biopsies from the colon and ileum of healthy subjects and IBD patients. Statistical significance of indicated comparisons using the Kruskal–Wallace test. For IHC, 10 samples were stained per group and a single representative image is shown at 20× magnification. Scale bar, 100 μM. *p < 0.05, **p < 0.01, ***p < 0.001. –infl, uninflamed; +infl, inflamed.

Previous work has shown that ICAM-1 and VCAM-1 have low expression whereas MAdCAM-1 is highly expressed on vascular endothelium in normal intestine (12–14). Although changes in patterns of expression have been described in IBD, shifts in overall gene expression have not been extensively evaluated. We therefore measured ICAM-1, VCAM-1 and MAdCAM-1 gene expression levels in healthy subjects and IBD patients. Consistent with historical protein data, we observed low but detectable levels of adhesion molecules in colonic and ileal biopsies from healthy subjects (Fig. 1A, 1C, 1E). ICAM-1 was highly upregulated in inflamed tissue from both UC and CD patients in comparison with uninflamed and healthy control tissue in both ileum and colon (Fig. 1A). In contrast, VCAM-1 gene expression was only moderately increased in inflamed CD, but not UC, samples (Fig. 1C). MAdCAM-1 upregulation was observed in both inflamed UC and CD tissue (Fig. 1E). We found no difference in gene expression between uninflamed UC or CD and healthy subject samples for any of the three adhesion molecules (Fig. 1A, 1C, 1E).

Because migration patterns change during inflammation, gene expression changes may reflect differences in expression between distinct cellular compartments. Inflamed biopsies from both ileum and colon exhibited consistent increases in ICAM-1 gene expression (Fig. 1A) and widespread upregulation of ICAM-1 protein (Fig. 1B) compared with uninflamed biopsies and controls (Supplemental Fig. 1A, 1B). These findings are consistent with previous reports demonstrating ICAM-1 expression on monocytes and plasma cells as well as endothelial cells in inflamed tissue (28). VCAM-1 protein levels were less altered (Fig. 1D, Supplemental Fig. 1C, 1D). MAdCAM-1 staining showed increased staining limited to endothelial cells (Fig. 1F, Supplemental Fig. 1E, 1F), consistent with previous reports (32).

### Expression of β1 and β7 integrins is unchanged on peripheral blood T cell populations in IBD

To better understand changes in integrin expression on lymphocytes from patients undergoing intestinal resection because of UC, CD, and diverticulitis (non-IBD), we evaluated β1 and β7 integrin expression on the surface of peripheral blood T cells.

**FIGURE 2.**
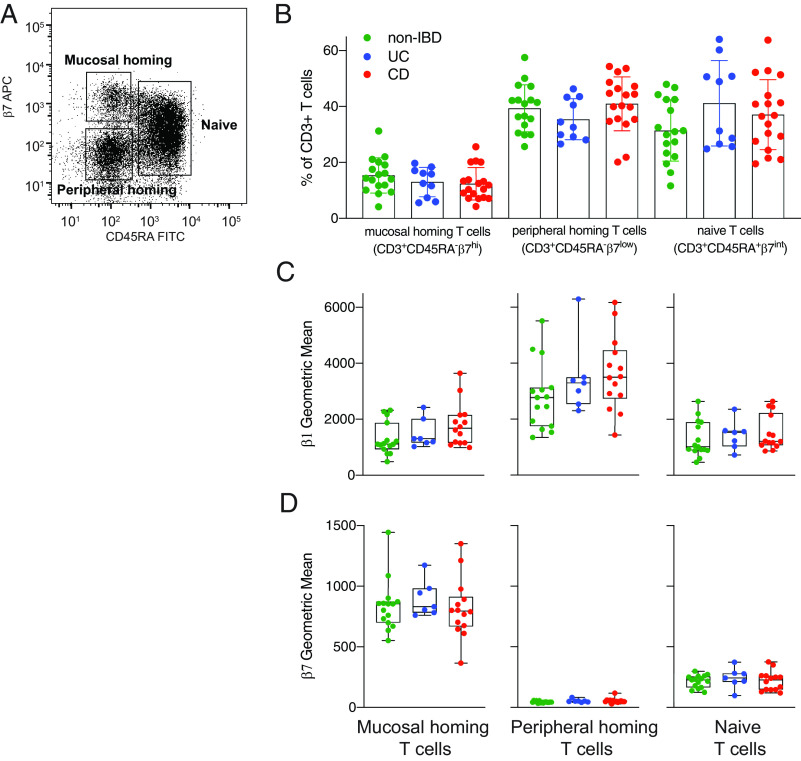
Peripheral T cells express both β1 and β7 integrins. Peripheral blood T cells from patients with UC, CD, and diverticulitis (non-IBD) undergoing intestinal resection from the Mayo cohort were evaluated for integrin expression. (**A**) Peripheral blood CD3^+^ T cells were gated into CD45RA^+^ and CD45RA^low^ populations, with the CD45RA^low^ population further gated into β7^high^ and β7^low^ subsets. (**B**) The frequency of mucosal homing (CD45RA^low^ β7^high^), peripheral homing (CD45RA^low^ β7^low^), and naive (CD45RA^+^) T cells in peripheral blood in non-IBD, UC, and CD patients undergoing intestinal resection. (**C**) β1 integrin expression and (**D**) β7 integrin expression on (left to right) mucosal homing, peripheral homing, and naive peripheral blood T cell subsets.

Peripheral blood T cells were separated into three groups based on high surface expression of CD45RA (CD45RA^+^; naive T cells), low surface expression of CD45RA and high β7 integrin (CD45RA^–^β7^high^; mucosal homing T cells) or low β7 integrin expression (β7^low^; peripheral homing T cells) (example of gating strategy is shown in (Fig. 2A). The frequency of these different subpopulations in peripheral blood was similar between UC and CD and between IBD and non-IBD groups (Fig. 2B).

Expression of β1 and β7 integrins was observed on all T cell subsets with the exception of β7 integrin on peripheral homing T cells (Fig. 2C, 2D). On mucosal homing T cells, both β1 and β7 integrin levels were similar between all patient groups (Fig. 2C, 2D). β1 expression was 3-fold higher on peripheral blood homing T cells in comparison with naive and mucosal homing T cells (Fig. 2C), whereas β7 expression was 4-fold higher on average on mucosal homing T cells in comparison with naive T cells (Fig. 2D). Similar findings were observed for all subsets for both CD4^+^ and CD8^+^ T cells, with the exception of β1 expression, which was only 2-fold higher on peripheral blood homing CD8^+^ T cells in comparison with naive and mucosal homing cells CD8^+^ T cells (Supplemental Fig. 2).

### Induction of αE integrin on T cell populations through TGF-β1 stimulation is not dependent on baseline β7 expression

**FIGURE 3.**
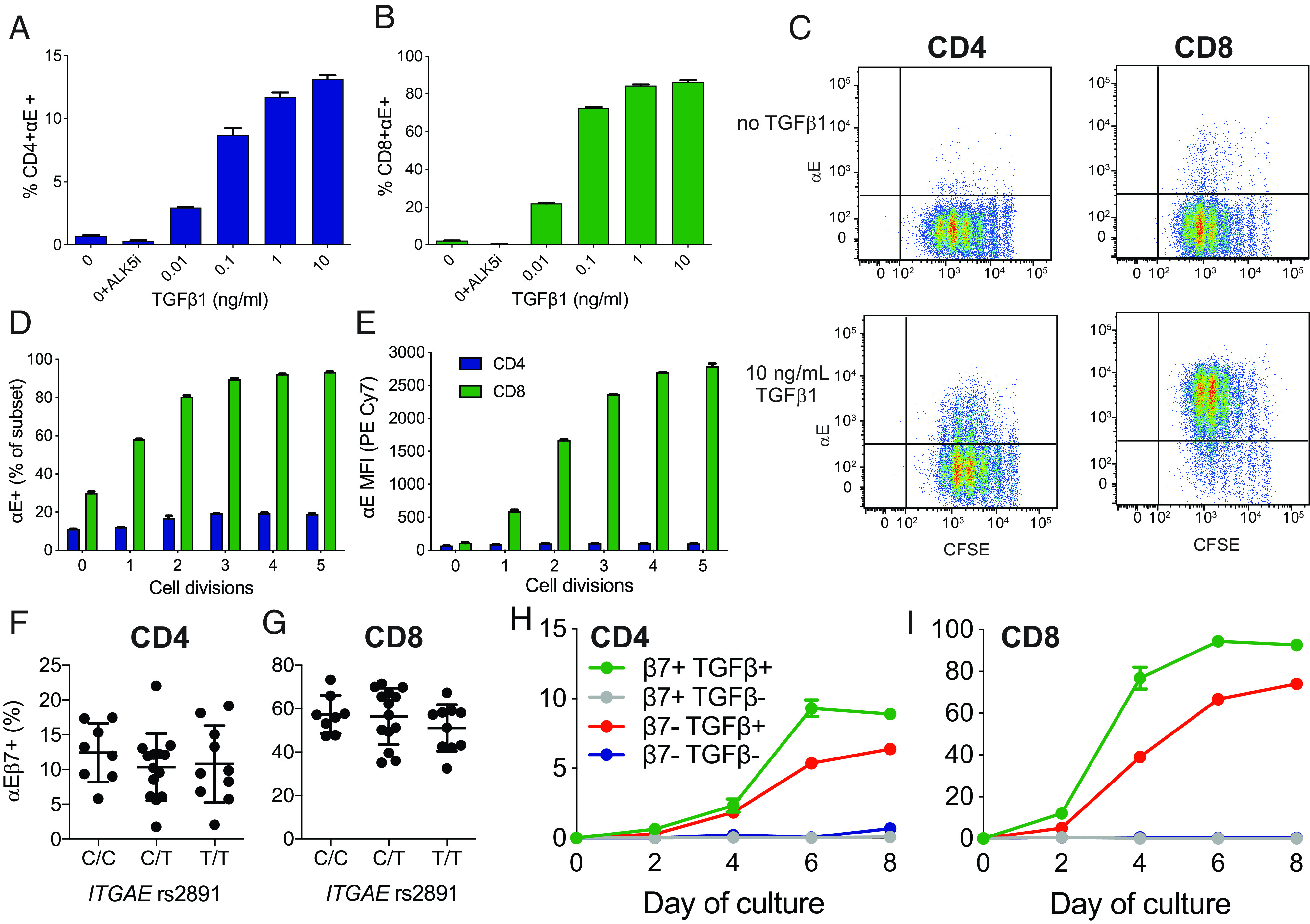
αE integrin can be induced by TGF-β1 and anti-CD3/CD28 stimulation of peripheral blood T cells independent of baseline β7 integrin expression. Peripheral blood T cells from healthy donors were stimulated for 4–9 d with anti-CD3/CD28 ± TGF-β1. Increasing cell surface expression of αE integrin was observed on (**A**) CD4^+^ and (**B**) CD8^+^ peripheral blood T cells in response to increasing doses of TGF-β1 after 9 d of culture. Data are representative of two independent experiments. No αE cell surface expression was found on either T cell population when ALK5 inhibitor was added to the culture. (**C**) CFSE dilution at 4 d shows increased αE expression on CD4^+^ and CD8^+^ T cells with increasing cell division in the presence of TGF-β1. Increases in both the (**D**) overall percentage of αE^+^ T cells and the (**E**) MFI of αE on both CD4^+^ and CD8^+^ T cells. Data in (C)–(E) are representative of three independent experiments. (**F** and **G**) PBMCs from *ITGAE* rs2981 genotyped healthy subjects with either C and/or T alleles were stimulated with anti-CD3/CD28/TGF-β1 for 6 d, then CD4^+^ and CD8^+^ T cells were evaluated for αE expression. (**H** and **I**) Peripheral blood T cells were sorted by baseline expression of β7 integrin and stimulated with anti-CD3/CD28 ± TGF-β1 (10 ng/ml) for 8 d. αE integrin expression was observed following TGF-β1 stimulation on both β7^−^ and β7^+^ CD4^+^ and CD8^+^ T cells. Data are representative of three independent experiments.

The αEβ7 integrin is expressed by a very low proportion of peripheral blood T lymphocytes. T cell expression of αE integrin is known to be TGF-β inducible (30–32), as was confirmed in our experiments (Fig. 3). Both CD4^+^ and CD8^+^ T cells responded to increasing concentrations of TGF-β1 with increasing frequency of surface αE expression (Fig. 3A, 3B). A dose-response relationship was observed for αE induction, with a higher frequency of CD8^+^αE^+^ T cells at all doses tested in comparison with CD4^+^αE^+^ T cells. Treatment with an ALK5 (TGF-β receptor I kinase) inhibitor demonstrated that αE upregulation in both CD4^+^ and CD8^+^ T cells required TGF-βRI signaling (Fig. 3A, 3B). CFSE dye was used to follow αE upregulation in dividing CD4^+^ and CD8^+^ T cells following stimulation with anti-CD3/CD28 and 10 ng/ml TGF-β1 (Fig. 3C). A lower overall percentage of CD4^+^ T cells underwent αE upregulation, yet achieved maximal frequency of αE positivity more rapidly than CD8^+^ T cells (Fig. 3D). αE expression levels were also higher on CD8^+^ T cells and continued to increase over later cell divisions, whereas CD4^+^ T cells had lower MFIs, yet achieved these expression levels at earlier cell divisions (Fig. 3E). αE MFI on CD4^+^αE^+^ lymphocytes were not dependent on the number of cell divisions, whereas αE MFI of CD8^+^αE^+^ T cells increased with successive cell division (Fig. 3E). A similar level of upregulation of αE in response to anti-CD3/CD28 and 10 ng/ml TGF-β1 was observed on both CD4^+^ and CD8^+^ T cells from peripheral blood of IBD patients (Supplemental Fig. 3A). No effect on αE induction was observed when therapeutically relevant anti-integrin Abs (anti-α4β7 or anti-β7) were included in the culture for the duration of the stimulation (Supplemental Fig. 3B).

TGF-β1–driven induction of αE expression on CD4^+^ T cells has been reported to be reduced in sarcoidosis patients carrying a genetic polymorphism in the promoter of the *ITGAE* gene tagged by the rs2981 single nucleotide polymorphism (22). We evaluated reported genetic risk alleles in the *ITGAE* gene in genotyped healthy donors to assess effects on surface αE expression. In contrast to previous studies, we found no association between rs2981 and αE expression by frequency or surface levels in either CD4^+^ (Fig. 3F) or CD8^+^ T cells (Fig. 3G). We also observed no significant differences in αE expression by frequency or surface levels for described intronic and exonic alleles (data not shown).

Although TGF-β1 can induce αE expression on both CD4^+^ and CD8^+^ T cells, the baseline requirement for β7 expression prior to upregulation of αE has not been extensively evaluated (33). We sorted T cells by β7 expression and CD4 or CD8 lineage prior to stimulation with anti-CD3/CD28 and TGF-β1. Following in vitro stimulation, both β7^+^ and β7^−^ T cells upregulated αE expression (Fig. 3H, 3I). Both β7^+^ and β7^–^ CD4^+^ and CD8^+^ T cells upregulated αE expression in response to TGF-β1 by day 6–8, with ∼20% relatively fewer αE^+^ cells observed in baseline β7^–^ cells (Fig. 3H, 3I). These data confirm that both β7^−^ CD4^+^ and CD8^+^ T cells can be induced to upregulate αE following TGF-β1 stimulation in vitro, although the frequency of cells with inducible αE expression is consistently lower than for cells that already have surface β7 expression.

### pSMAD3 and increased TGF-β–inducible gene expression in IBD intestinal mucosa

TGF-β regulates transcription of the αE and β7 integrin genes (*ITGAE* and *ITGB7*, respectively) (33–35). Serine/threonine phosphorylation of SMAD2 and SMAD3 following TGF-β activation of ALK5 (TGF-βR1) as well as NFAT-1 activity have been shown to drive expression of *ITGAE* (36, 37). Indeed, both NFAT-1 and SMAD binding sites have been identified in the *ITGAE* promoter and enhancer (36). The *ITGB7* promoter contains two functional TGF-β response elements (35). Conversely, TGF-β leads to reduced transcription of the α4 integrin subunit (35).

**FIGURE 4.**
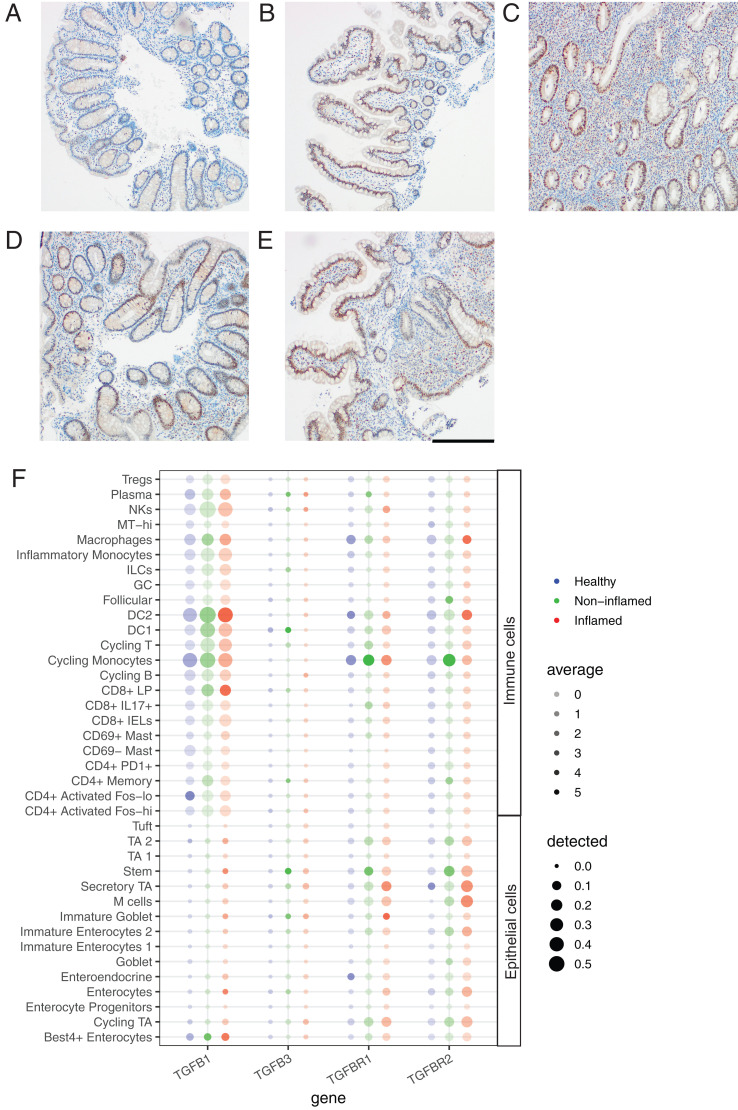
Inflamed colonic tissue exhibits increased pSMAD3 with TGF-βR expression on immune and epithelial cells. Intestinal biopsies from healthy subjects or IBD patients from the Newcastle cohort were evaluated for evidence of TGF-β–induced signaling or gene expression. pSMAD3 expression in the colon and ileum during active IBD. pSMAD3 (DAB: brown) staining is shown for (**A**) colon and (**B**) ileum for matched tissue biopsies from a healthy subject. Actively inflamed colonic biopsies from a (**C**) UC and (**D**) CD patient. (**E**) Representative image of an inflamed ileal biopsy from a CD patient. Images are representative of staining from five subjects in each group. Images are shown at 10× magnification. Scale bar, 200 μM. (**F**) Expression of TGF-β1, TGF-β3 and their receptors was evaluated in single cell RNA-sequencing data from healthy volunteers (*n* = 12) and IBD patients (*n* = 18). Upregulation of TGF-βR1 and TGF-βR2 was observed in inflamed colonic tissue.

We used IHC to evaluate changes in SMAD3 phosphorylation (pSMAD3) in biopsies from IBD patients in comparison with healthy subjects. In healthy subjects, pSMAD3 was largely limited to the epithelium in both the colon (Fig. 4A) and ileum (Fig. 4B), with a few scattered mononuclear cells also staining positive. The majority of epithelial cells displayed positive staining for pSMAD3 in the ileum, with a lower frequency of positive cells in the colon. pSMAD3^+^ mononuclear cells were also more frequent in the ileum than in the colon of healthy subjects. Colonic biopsies from UC patients with active disease showed increased infiltration of mononuclear cells, many of which were pSMAD3^+^ (Fig. 4C). Epithelial staining for pSMAD3 in UC biopsies was more intense than in healthy colonic tissue. Similarly, colonic biopsies from CD patients showed increased epithelial staining intensity (Fig. 4D). Ileal biopsies from CD patients had localized increases in pSMAD3^+^ mononuclear cells in inflamed areas (Fig. 4E) with overall similar intensity of pSMAD3 in the epithelium compared with healthy ileum (Fig. 4B).

Analysis of TGF-β family members and receptor expression in isolated cells using single-cell RNA-sequencing data (38) showed that TGF-β1 is the predominant family member expressed in the colon and is expressed primarily by immune cells, whereas TGF-β receptor expression was found on both immune and epithelial cells (Fig. 4F). TGF-β3 was detected at low levels whereas TGF-β2 was not detectable. Expression of TGF-β1 and both TGF-β receptors was similar between healthy, inflamed, and uninflamed biopsies. TGF-β induces a much broader gene expression program beyond *ITGAE* and *ITGB7*, including common and distinct elements in different cell types (39). A common gene induced across cell types is EGR1, a transcription factor (37) with binding sites near TGF-β1 target genes, including *SMAD7*, *ID1*, and *SKIL* (40). Expression of the TGF-β–inducible genes EGR1 and EGR2 in intestinal biopsies from healthy subjects and IBD patients (Supplemental Fig. 3E, 3F). Increased gene expression of both EGR1 and EGR2 was observed in inflamed colonic biopsies versus healthy colon or uninflamed biopsies from IBD patients. There was no significant difference in EGR1 or EGR2 gene expression in ileal biopsies, consistent with the stronger pSMAD3 staining observed in the colon in comparison with the ileum.

### Gene expression in induced αE^+^ T cells is independent of baseline β7 expression status

**FIGURE 5.**
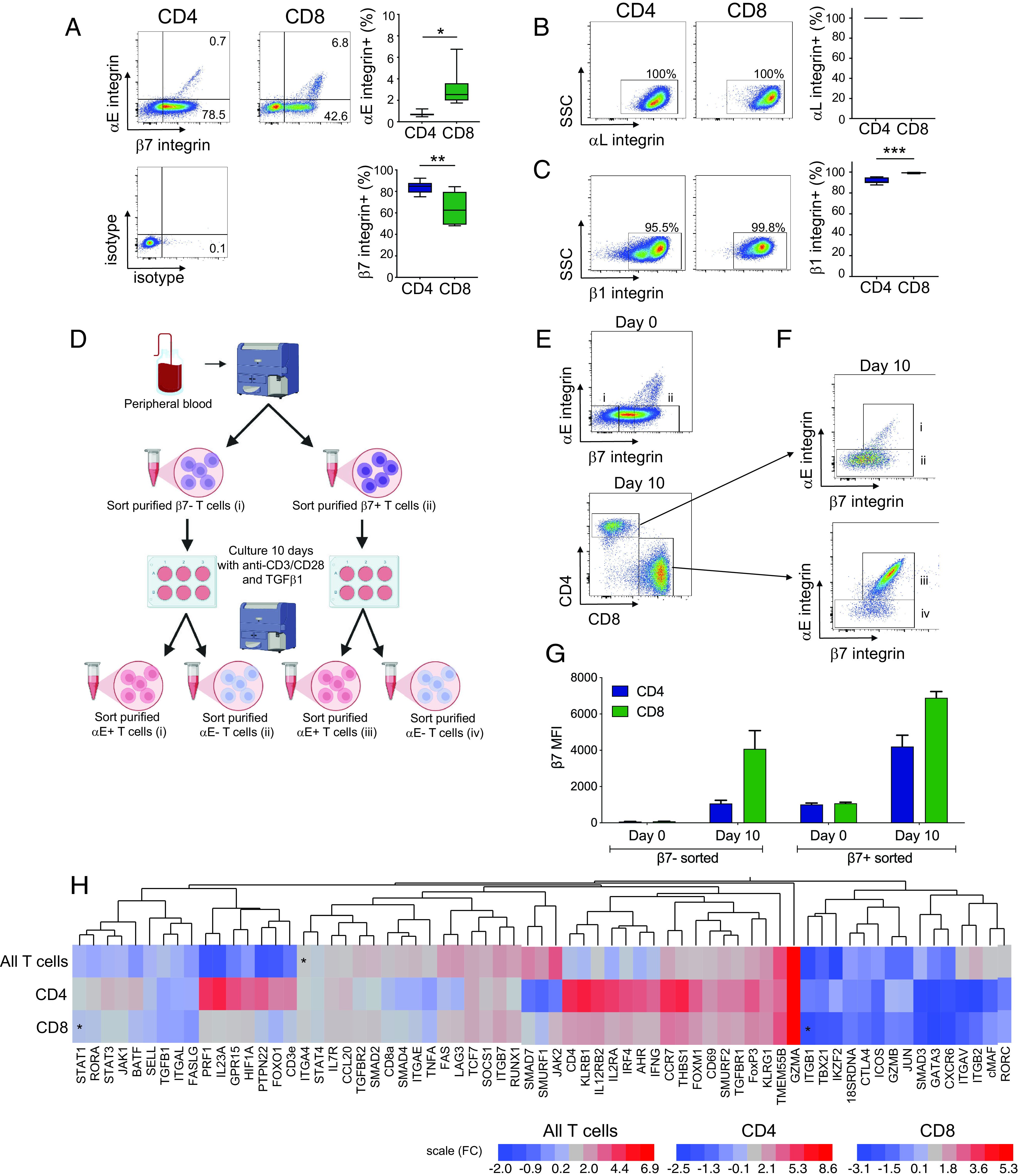
Baseline β7 expression does not drive differences in gene expression in αE^+^ and αE^−^ T cells. (**A**) PBMCs were obtained from seven healthy donors and evaluated for surface expression of (A) αE and β7 (**B**) αL and (**C**) β1integrin subunits. Representative FACS plots of CD3^+^CD4^+^ and CD3^+^CD8^+^ T cell expression are shown with accompanying isotype control staining and Tukey plots of the percentage of CD4^+^ and CD8^+^ cells that express each integrin subunit. (**D**) Culture strategy for evaluation of the effect of baseline β7 integrin expression on αE induction and gene expression, showing final groups in analysis. (**E**) PBMCs were sort purified into 1) β7^−^ and 2) β7^+^ fractions, then stimulated for 10 d with anti-CD3/CD28 plus TGF-β1 for 10 d. (**F**) After 10 d of culture, four cell populations 1) CD4^+^αE^+^, 2) CD4^+^αE^–^, 3) CD8^+^ αE^+^, and 4) CD8^+^αE^−^ were sorted from baseline β7^−^ and from baseline β7^+^ cultures, yielding eight total groups for gene expression analysis from each of the seven donors. (**G**) Baseline and day 10 levels of β7 surface expression were measured and increased β7 was observed in all groups. (**H**) Fold change of candidate genes (cytokines, chemokines, cytokine, and chemokine receptors, signaling molecules, transcription factors, T cell surface markers, and effector molecules) is shown. Significant differences are indicated by an asterisk.

pSMAD3 levels in the inflamed colon suggest that TGF-β signaling is increased in active colonic disease. We hypothesized that upregulation of αE integrin by local active TGF-β would not require pre-existing expression of the α4β7 dimer and that both β7^−^ and β7^+^ peripheral blood T cells can induce αE following migration into the intestinal mucosa. To evaluate potential differences in the αE^+^ T cell phenotype due to initial β7 integrin expression, we developed a two-step in vitro culture and cell sorting approach to enable comparison of gene expression between these populations. Data from seven donors confirmed expression of αE integrin on <1% of peripheral blood CD4^+^ T cells and ∼3% of peripheral blood CD8^+^ T cells, with 80% of CD4^+^ T cells and 60% of CD8^+^ T cells showing high expression of β7 integrin (Fig. 5A). Ubiquitous expression of αL integrin was observed on peripheral CD4^+^ and CD8^+^ T cells (Fig. 5B). β1 integrin was observed on ∼90% of CD4^+^ T cells and nearly all CD8^+^ T cells (Fig. 5C). Initial cell sorting was based on β7 expression, with low/negative β7 (peripheral homing) and high β7 (mucosal homing) T cells sorted from seven healthy donors (Fig. 5D, 5E). Following 10 d of culture with anti-CD3/CD28 beads and 10 ng/ml TGF-β1, a subset of T cells had upregulated αE surface expression. Higher induction of αE was again observed on CD8^+^ in comparison with CD4^+^ cells, and cells were sorted into αE^+^CD4^+^, αE^−^CD4^+^, αE^+^CD8^+^, and αE^−^CD8^+^ fractions (Fig. 5F). Consistent with upregulation of αE surface expression in response to TGF-β1, there was a clear increase in β7 MFI in all cell groups over the 10 d of culture (Fig. 5G).

Analysis using a linear model approach to evaluate the effect of baseline β7 expression was performed independently for CD4^+^ and CD8^+^ T cells using αE surface expression at day 10 as a covariate (Fig. 5H). T cell lineage genes, TGF-β1 response genes, and genes associated with αEβ7 integrin (18, 20) were selected for evaluation based on expression in cell types of interest. For CD4^+^ T cells, baseline β7 integrin expression had a nominally significant (unadjusted *p* < 0.05) effect on the following genes: ITGA4, GATA3, GZMA, BATF, HIFA, ICOS and IRF4, although none of these genes had an FDR below 0.1. For CD8^+^ T cells, STAT1, ITGB1, ITGA4, CD127, and CD4 demonstrated a significant (*p* < 0.05) effect of baseline β7 integrin expression. Both STAT1 and ITGB1 had an FDR below 0.05. The majority of genes showed no difference in expression related to initial β7 expression levels.

## Discussion

Inflammatory events have the potential to change established patterns of cellular migration and lymphocyte differentiation in patients with IBD. Under homeostatic conditions, lymphocytes activated within mucosal lymph nodes to express high levels of α4β7 integrin as well as naive cells with intermediate α4β7 integrin expression circulate through the intestinal mucosa. In IBD, activation of inflammatory cascades can alter expression patterns of adhesion molecules that serve as entry points into mucosal tissues. Increased levels of adhesion molecules on the intestinal endothelium may alter lymphocyte migration into the gut. We evaluated VCAM-1, ICAM-1, and MAdCAM-1 gene and protein expression in healthy mucosa from normal subjects and uninflamed and inflamed mucosa from patients with IBD. Although inflamed mucosa had generally increased expression of adhesion molecules, ICAM-1 was broadly upregulated, consistent with its known expression by infiltrating monocytes and plasma cells, whereas VCAM-1, and in particular MAdCAM-1, were more restricted in expression to endothelial cells. Subsets of peripheral blood T cells can express both α4β7 and/or α4β1, consistent with previous data showing that T cells express multiple integrins for entry into mucosal tissues (20). Once cells have migrated into the mucosa, induction of other integrins can occur. We demonstrate that T cells without α4β7 expression can be induced to express αE integrin following exposure to TGF-β1 under activating conditions. Downstream TGF-β gene expression and pSMAD3, a key part of the TGF-β signaling pathway, are both increased in the colon of IBD patients in comparison with healthy subjects, suggesting a TGF-β rich environment. TGF-β1 was the predominant TGF-β family member by gene expression in colonic macrophages, T cells and DCs, whereas TGF-βR1 and TGF-βR2 are expressed on monocytes, T cells and epithelial cells. Finally, following the induction of αE expression on T cells that were either α4β7^+^ or α4β7^−^ by TGF-β1, gene expression was largely not dependent on initial β7 expression. This suggests that T cells that undergo α4β7-independent migration into mucosal tissue sites can give rise to inflammatory αE^+^ T cells and therefore that α4β7-directed therapy alone may be insufficient to modulate αEβ7^+^ lymphocyte retention in vivo. How frequently this occurs in the human gut cannot be measured using current clinical techniques.

IBD may increase the likelihood that both α4β7^+^ and α4β7^−^ T cells enter the intestinal mucosa through compensatory homing mechanisms. Increased expression of ICAM-1 and VCAM-1 (Fig. 1), which has been shown to resolve at the gene expression level following anti-TNF treatment (41), may enable entry of these cells into the intestinal mucosa in IBD. Our data in healthy subjects were from two age-matched cohorts and showed increased expression of adhesion molecules at the gene expression and protein level. We found that peripheral blood T cells coexpress β1 and α4β7 on a significant fraction of mucosal homing T cells and naive T cells (Figs. 2 and (5). Previous reports show an increase in peripheral blood α4^+^β1^high^ cells and a corresponding increase in α4^+^β1^high^ T cells in intestinal mucosa from CD patients (7). In the absence of a human Ab specific for the α4β1 dimer, it remains to be determined whether α4β1 is expressed on the cell surface of circulating cells, or whether β1 is paired with another of the 10 α-integrin chains that are known to form β1 heterodimers. Experiments using humanized mice and mouse models show that lymphocytes use compensatory homing mechanisms to enter the mucosa; this process can be blocked using a small molecule inhibitor specific for α4β1 (7).

Once T cells have entered the LP, TGF-β1 can induce upregulation of αE through SMAD phosphorylation. Our data suggest that TGF-β–induced gene expression may occur more readily upon entry into the colon than the ileum during active IBD where increases in pSMAD3 and TGF-β–inducible genes were observed. Previous work has shown that colonic αE^+^CD4^+^ T cells express increased inflammatory cytokines such as IL-17 and IFN-γ (21). The frequency of αE^+^CD4^+^ T cells is increased in UC, which is consistent with our observations of increased TGF-β signaling. When we induced αE expression by treating activated T cells in vitro, we detected few gene expression differences for inflammatory cytokines or effector molecules between cells that were originally α4β7^+^ and α4β7^−^. These data suggest that cells that migrate into the mucosa using compensatory homing mechanisms can develop an αE-associated inflammatory phenotype. Abs that block αE integrin suppress accumulation of inflammatory lymphocytes in the intestinal mucosa (9, 24), indicating that once αEβ7 is induced, it increases cellular retention in the mucosa. The decrease in cellular accumulation was more pronounced at later time points in a humanized mouse model, consistent with effects on retention rather than initial homing (24).

Recent studies have specifically addressed migration of cells into tissue, which is challenging to directly evaluate in clinical studies. The effect of vedolizumab on lymphocyte trafficking was recently described in a study that followed labeled cells using imaging, but observed no change in T cell migration to the intestinal mucosa (42). Flow cytometry within the same study protocol also failed to demonstrate a change in T cell frequency following 14 wk of vedolizumab treatment, supporting the idea that alternative migratory pathways following treatment with vedolizumab may be active. A separate study observed decreased α4β7^+^ within intestinal T cell subsets but no change in αEβ7^+^ T cells in the colon, whereas also noting incomplete occupancy on intestinal CD8^+^ T cells (43). Our in vitro differentiation studies show that initial β7 expression is not required for upregulation of αE. Indeed, patients treated with vedolizumab have been found to have an increase in αE^+^ CD8^+^ T cells in peripheral blood during maintenance time points, suggesting that blockade of α4β7 does not impair αE upregulation (43). Taken together, these findings suggest that blockade of α4β7 alone may not be sufficient to inhibit T cell trafficking to the intestinal mucosa, and β7^−^ negative cells may undergo induction of αEβ7 once in the mucosa, increasing retention of inflammatory T cells.

Inhibition of lymphocyte trafficking has proven to be an effective strategy for treating both UC and CD, yet in a significant fraction of patients, medication fails to induce and maintain remission. Although therapies designed to inhibit migration through blockade of α4β7 or MAdCAM-1 have shown efficacy, studies showing that blockade of α4β7/MAdCAM-1 interactions alone may leave additional homing and retention pathways intact. Consistent with this, studies evaluating changes in inflammatory gene expression following treatment with anti-integrins show that few genes are significantly changed by vedolizumab (anti-α4β7) treatment in comparison with inhibition of TNF-α at early time points in UC patients (44). Our more recent phase 3 study showed that etrolizumab significantly decreases lymphocyte gene expression at week 14 in CD patients (9), consistent with data from a phase 2 study of etrolizumab in UC (18). Continued migration of lymphocytes through α4β7-independent compensatory mechanisms and/or increased retention of lymphocytes following αEβ7 upregulation might yield insights into patients who do not respond to vedolizumab treatment.

In conclusion, we demonstrate that αEβ7 can be induced on both α4β7^+^ and α4β7^−^ T cells following exposure to TGF-β1, and we show evidence for increased TGF-β activity in the colon of patients with active IBD. These data, in combination with an increase in adhesion molecules in active IBD, highlight the potential for increased compensatory migration mechanisms to the gut mucosa beyond α4β7/MAdCAM-1 in active inflammation that can result in αE^+^ T cell accumulation. Inflammatory gene expression in αE^+^ T cells was largely unchanged between T cell populations that were originally α4β7^+^ and α4β7^−^ T cells. These data suggest that IBD patients treated with Abs that block α4β7 or MAdCAM-1 may benefit from additional integrin and/or inflammatory cytokine blockades to further inhibit inflammatory cell migration to the gut mucosa.

## Supplementary Material

Data Supplement
