# Supplementary material for: Regulation and Role of αE Integrin and Gut Homing Integrins in Migration and Retention of Intestinal Lymphocytes during Inflammatory Bowel Disease
Source: J Immunol. 2021 Nov 1;207(9):2245–54. doi: 10.4049/jimmunol.2100220 (PMC8525869; doi:10.4049/jimmunol.2100220)
Supplement: Data Supplement [file JI_2100220.zip › JI_2100220_Supplemental_1.pdf]

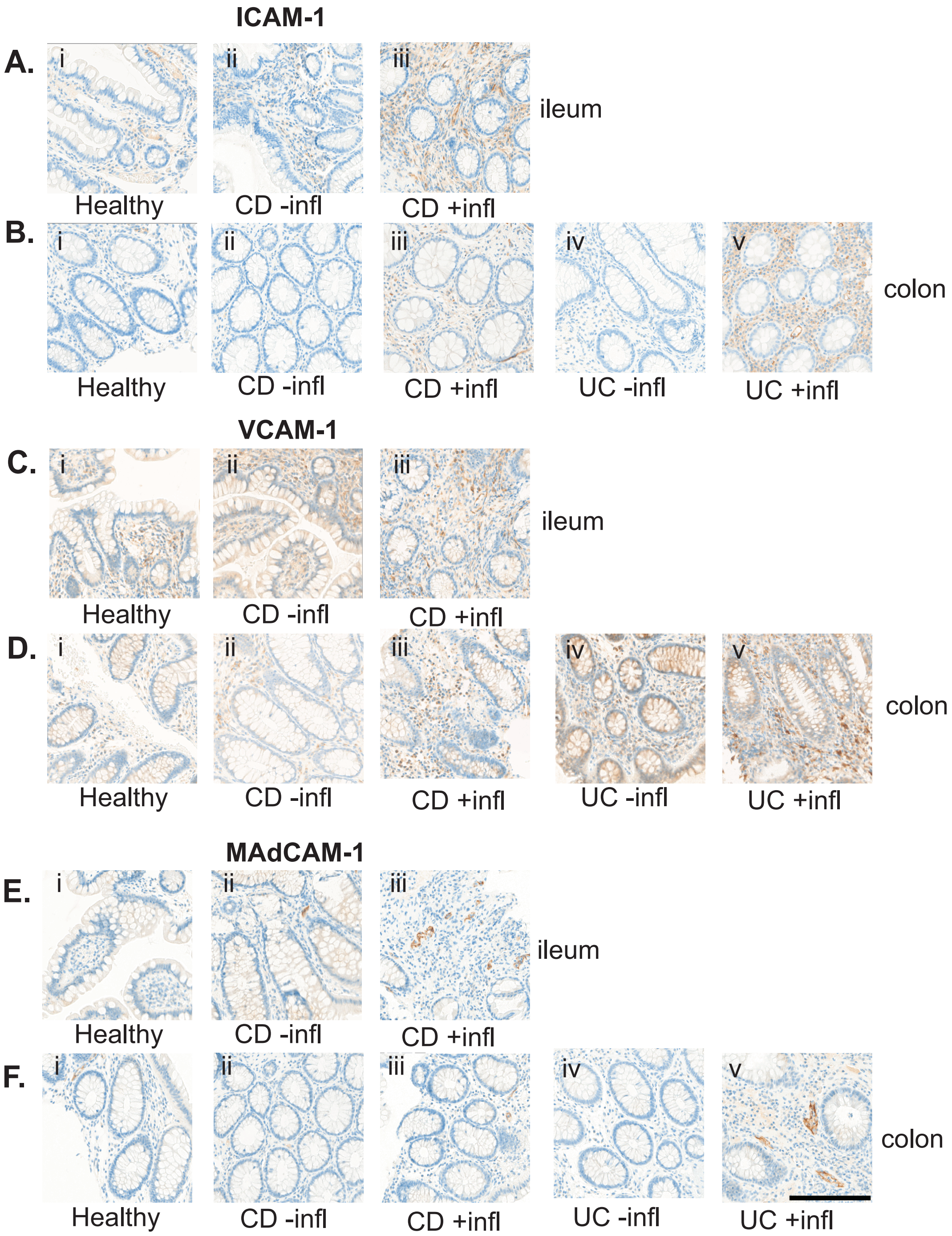

Supplemental Figure 1. Level of adhesion molecules ICAM-1, VCAM-1 and MAdCAM-1 in IBD. (A) Example of ICAM-1 staining from the ileum of (i) a healthy subject and (ii) uninflamed ileum and (iii) inflamed ileum from a patient with CD. **(B)** ICAM-1 staining of the colon of (i) a healthy subject, (ii) uninflamed and (iii) inflamed colon from a CD patient and (iv) uninflamed and (v) inflamed colon from a UC patient. **(C)** Example of VCAM staining from the ileum of (i) a healthy subject and (ii) uninflamed ileum and (iii) inflamed ileum from a patient with CD. **(D)** VCAM staining of the colon of (i) a healthy subject, (ii) uninflamed and (iii) inflamed colon from a CD patient and (iv) uninflamed and (v) inflamed colon from a UC patient. **(E)** Example of MAdCAM-1 staining from the ileum of (i) a healthy subject and (ii) uninflamed ileum and (iii) inflamed ileum from a patient with CD. **(F)** MAdCAM-1 staining of the colon of (i) a healthy subject, (ii) uninflamed and (iii) inflamed colon from a CD patient and (iv) uninflamed and (v) inflamed colon from a UC patient. Images shown at 20X magnification. Scale bar is 100 μM.

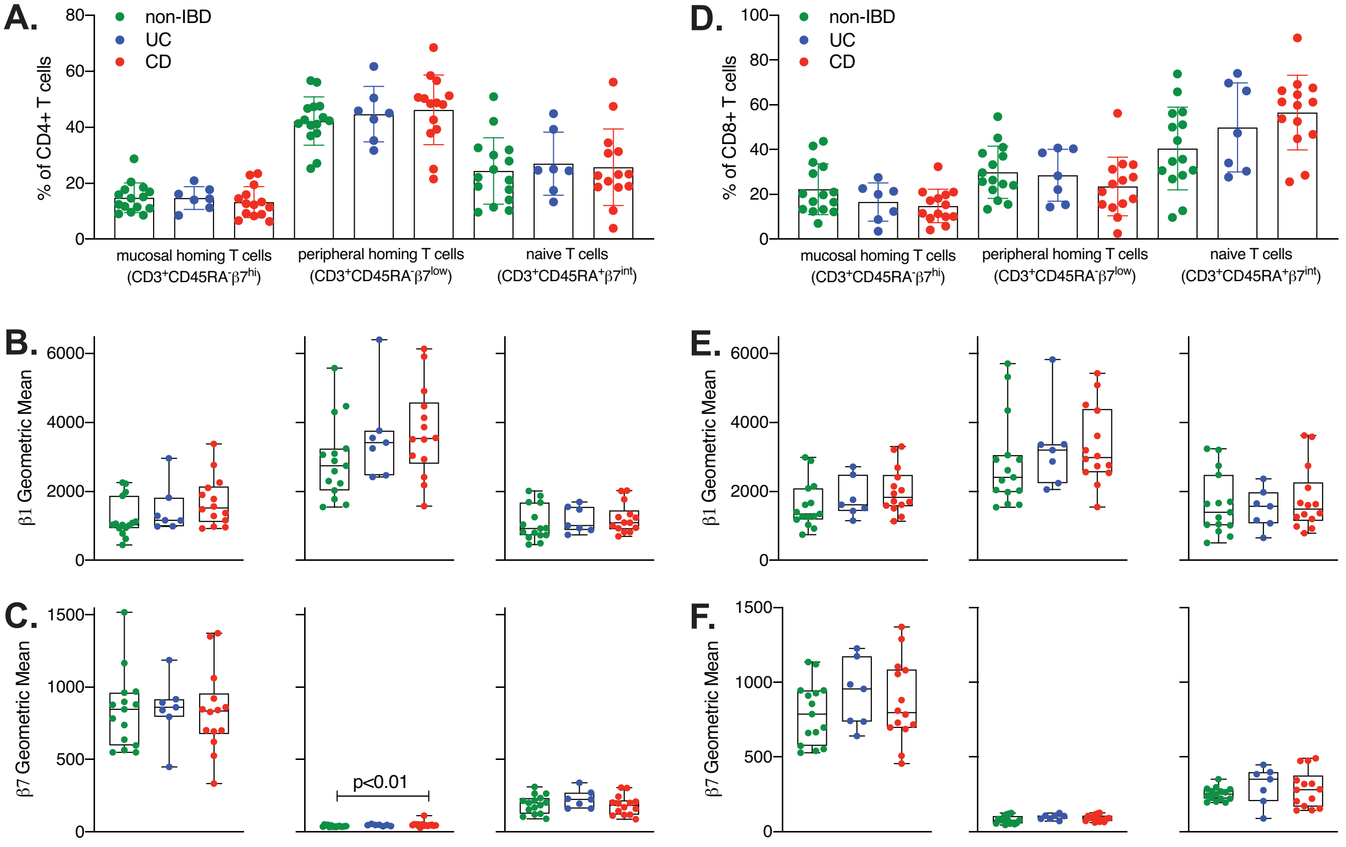

Supplemental Figure 2. Peripheral CD4 and CD8 T cells express both β1 and β7 integrins. Peripheral blood T cells from patients with UC, CD and diverticulitis (non-IBD) undergoing intestinal resection were evaluated for integrin expression. **(A)** Peripheral blood CD3+CD8- (CD4+) T cells were gated into CD45RA^{+} and CD45RA^{low} populations, with the CD45RA^{low} population further gated into β7^{high} and β7^{low} subsets. Geometric mean of **(B)** β1 integrin and **(C)** β7 integrin on (left to right) mucosal homing, peripheral homing and naive CD4+ T cell populations. **(D)** The frequency of CD3+CD8+ naïve, mucosal homing and peripheral homing T cells in peripheral blood. Geometric mean of **(E)** β1 integrin and **(F)** β7 integrin on (left to right) mucosal homing, peripheral homing and naive CD8+ T cell populations.

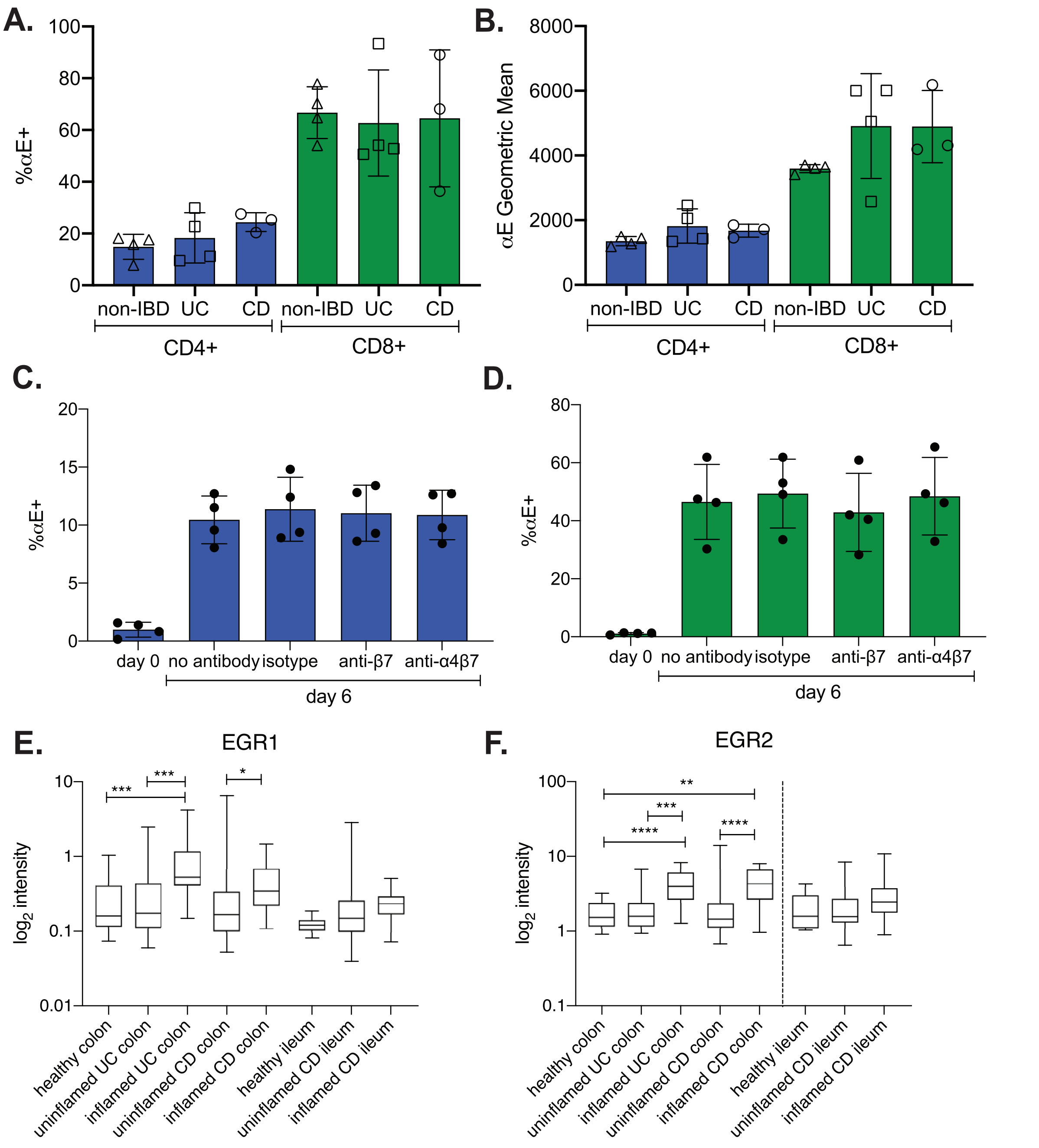

Supplemental Figure 3. αE upregulation is similar on T cells from IBD patients and non-IBD patients is not impaired by anti-integrins while inflamed colonic tissue exhibits increased TGF-β inducible gene expression. (A-B) Induction of αE expression on peripheral CD4+ and CD8+ T cells from UC (n=4), CD (n=3) and non-IBD (n=4) patients (Mayo cohort). Following a six-day culture of PBMCs with anti-CD3/CD28/TGF-β1, both **(A)** frequency and **(B)** surface levels of αE were measured on T cells. **(C-D)** Stimulation of PBMCs from four healthy volunteers with anti-CD3/CD28/TGF-β1 was done in the presence of anti-integrin antibodies. No effect on αE expression was observed in either **(C)** CD4+ T cells or **(D)** CD8+ T cells. **(E-F)** Expression of TGF-β inducible genes **(E)** EGR1 and **(F)** EGR2 was evaluated in biopsy samples from healthy volunteers, UC and CD patients in the EMBARK dataset. Upregulation of EGR1 and EGR2 was observed in inflamed colonic tissue. -infl, uninflamed; +infl, inflamed.

Supplemental Table 1. Clinical characteristics for IBD cohorts shown in Figure 1 B, D, F and 4A-E.

Newcastle (Figure 1 and Figure 4)	Healthy subjects (n=10)	UC patients		CD patients	
		Active (n=10)	Inactive (n=10)	Active (n=10)	Inactive (n=10)
Age (median, range), years	45<br>37-47	50<br>35-63.5	53.5<br>42-60	39<br>28-51	40<br>27-46
Sex (M/F)	6/4	6/4	3/7	5/5	3/7

EMBARK (Figure 1)	Healthy subjects (n=12)	UC patients		CD patients	
		Active (n=22)	Inactive (n=21)	Active (n=27)	Inactive (n=58)
Age (median, range), years	55<br>26-57	36<br>20-62	38<br>24-64	34<br>20-59	34<br>18-65
Sex (M/F)	4/8	13/9	10/11	13/14	26/32

Mayo (Figure 2)	Non-IBD subjects (n=15)	UC patients (n=9)	CD patients (n=18)
Age (median, range), years	60<br>45-78	61<br>30-72	40<br>20-75
Sex (M/F)	7/8	6/3	2/16
